# Circulating Tumor DNA as Biomarkers for Cancer Detection

**DOI:** 10.1016/j.gpb.2016.12.004

**Published:** 2017-04-07

**Authors:** Xiao Han, Junyun Wang, Yingli Sun

**Affiliations:** 1 CAS Key Laboratory of Genomic and Precision Medicine, China Gastrointestinal Cancer Research Center, Beijing Institute of Genomics, Chinese Academy of Sciences, Beijing 100101, China; 2 University of Chinese Academy of Sciences, Beijing 100049, China

**Keywords:** Precision medicine, Liquid biopsy, Circulating tumor DNA, Biomarker, Clinical diagnosis, Cell-free nucleic acids

## Abstract

Detection of **circulating tumor DNAs** (ctDNAs) in cancer patients is an important component of cancer **precision medicine** ctDNAs. Compared to the traditional physical and biochemical methods, blood-based ctDNA detection offers a non-invasive and easily accessible way for cancer diagnosis, prognostic determination, and guidance for treatment. While studies on this topic are currently underway, clinical translation of ctDNA detection in various types of cancers has been attracting much attention, due to the great potential of ctDNA as blood-based **biomarkers** for early diagnosis and treatment of cancers. ctDNAs are detected and tracked primarily based on tumor-related genetic and epigenetic alterations. In this article, we reviewed the available studies on ctDNA detection and described the representative methods. We also discussed the current understanding of ctDNAs in cancer patients and their availability as potential **biomarkers** for clinical purposes. Considering the progress made and challenges involved in accurate detection of specific **cell-free nucleic acids**, ctDNAs hold promise to serve as **biomarkers** for cancer patients, and further validation is needed prior to their broad clinical use.

## Introduction

Over 8.2 million people die of cancer each year due to the inaccessibility of appropriate detection procedures and treatments [1]. Researchers have been exploring methods for the detection and cure of cancers via cancer screening, prognostic determination, and monitoring. However, up till now, there are no known diagnostic methods that do not hurt the physical health of patients during the process of cancer detection. For example, radiology is extensively used in cancer detection, but excessive ionizing radiation could pose potential health risk to the examined patient [2], [3]. On the other hand, non-radiation modalities, such as ultrasound scans and magnetic resonance imaging (MRI) scans, are thought to be inefficient for the detection of minimal residual disease [4], [5], [6]. Furthermore, the “solid biopsy” method of detection is invasive, and cannot accurately track dynamic changes in tumors due to tumor heterogeneity [7], [8], [9]. Thus, developing non-invasive and precise methods for the early diagnoses of cancers is an increasingly urgent requirement in the era of precision medicine (PM).

Liquid biopsy is a type of technique for sampling and analyzing of non-solid biological tissues, mainly used in disease diagnosis [10]. Circulating tumor DNAs (ctDNAs), being a popular class of liquid biopsy biomarkers, are believed to be easily detected in the plasma of cancer patients even in the early stages of their disease [11], [12], [13]. ctDNAs display considerable variations in DNA sequences. Moreover, tumor-specific DNA methylations can also be consistently measured and reflected within ctDNAs, showing the potential for wide application in clinical detection of cancers [14], [15], [16], [17], [18]. To provide an overview of current utilities of clinical therapy and potential biomarkers, we summarized the methods of detection that are frequently used nowadays, such as imaging-based methods [19], [20], [21] and solid biopsies [22], [23], [24], [25] in Table 1. Biomarkers that are currently in use or under investigation in liquid biopsies are also shown in the table, including proteins [26], [27], [28], circulating tumor cells (CTCs) [7], [11], [29], ctDNAs [10], [30], [31], circulating cell-free RNAs [32], [33], [34], and exosomes [35], [36], [37]. In this article, we mainly discuss several new methods for using ctDNAs and ctDNA methylations in the early detection of cancers. The challenges and potential applications of ctDNA detection are also discussed in this review.

## Profiles of ctDNAs and CTCs

On January 9, 2016, the Chinese Academy of Sciences (CAS) announced its precision medicine initiative (CASPMI). This initiative aims to establish a new medical paradigm characterized by high-efficiency and low-cost disease diagnoses and treatments of individual patients, based on their genetic and epigenetic composition. In this program, some studies would focus on the risks of occurrence of cancers and other major chronic diseases for early warning signs and interventions. Performing liquid biopsies, specifically by capturing CTCs and ctDNAs in the plasma or serum of cancer patients, is an ideal strategy for clinical utility in the PM research programs [38], [39].

For around 1000 years, biopsies have been used clinically for the diagnoses, management, and planning the treatments of diseases [10]. Given the many obstacles in sequentially obtaining repeated biopsies, including the inconvenience to the patients, the potential surgical complications, and the clinical risks, clinical use of multi-site biopsies is often impractical [22], [23], [24], [25]. As an alternate, liquid biopsies are currently being used to address the temporal and spatial heterogeneity in solid tumors. Liquid biopsies could even be used in cancer detection, thus facilitating early diagnoses and treatments [10], [40], [41], [42].

CTCs are shed into the bloodstream by primary tumors during early tumorigenesis [43]. They can be purified from blood, and separated from normal blood cells by the differences in their physicochemical characteristics [43]. Ashworth demonstrated the presence of CTCs in 1869 [44], but their value was overlooked until the 1990s [29]. The CTCs have immense potential for cancer detection and management of advanced disease, as reported in cases of breast cancers, prostate cancers, and colorectal cancers [45], [46], [47]. However, it is difficult to identify and isolate CTCs since they are present in circulation at the rate of only one CTC per 1 × 10^9^ normal blood cells in patients with metastatic cancers [48]. Many new approaches have lately been designed to select and capture CTCs due to the recent technological advances. The semi-automated CellSearch (Veridex) system is the most commonly used selection technique for CTC detection. It enriches cells that express epithelial-cell adhesion molecule (EpCAM) but lack expression of the leukocyte-specific molecule, cluster of differentiation 45 (CD45) [49]. Several microfluidic devices have been developed for CTC capture, including the CTC-chip (based on microfluidic and chip technology), micro-Hall detector, and CTC-iChip (an inertial focusing-enhanced microfluidic CTC capture platform) [50], [51], [52].

The CTC-based liquid biopsy assays have high specificities. In addition, they also display low signal-to-noise ratios, particularly in the detection of early-stage disease. Compared to CTC detection, the ctDNA assay can provide personalized disease detection, and is disease- and treatment-specific for individual patients. CtDNA can provide a personalized snapshot of the patient’s disease status. Additionally, ctDNA is likely to exhibit increased sensitivity for early detection of cancers. Unlike CTC capture, ctDNA enrichment does not depend on the use of special equipment [7].

**Figure 1:**
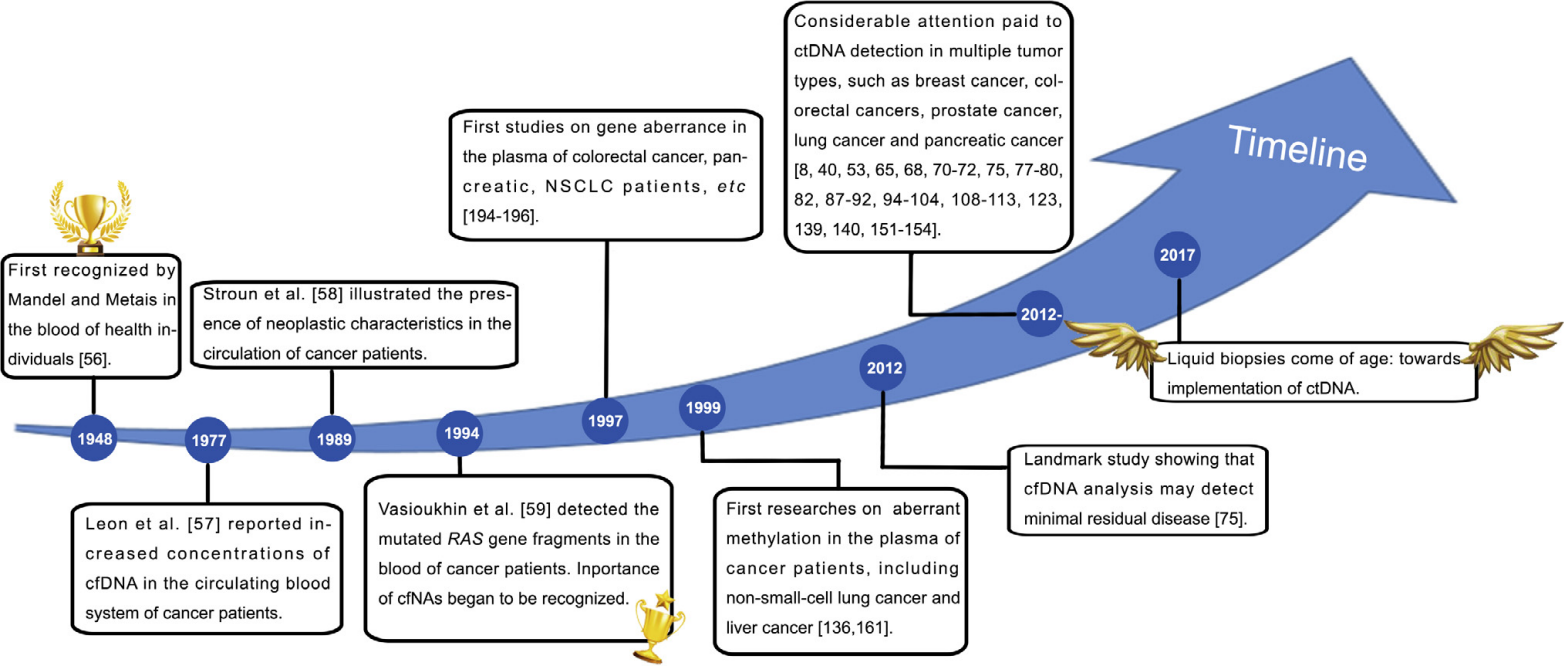
**Landmarks in the detection of ctDNAs in patients with different cancers** This timeline shows the development of ctDNA detection of genetic and epigenetic alterations. Since the first validation of ctDNAs in 1948 [56], increasing interest has been attracted due to its ability for detection and broad clinical applicability. In 1977, Leon et al. [57] found increased concentrations of cfDNAs circulating in cancer patients. Ten years later, Stroun et al. [58] illustrated the presence of neoplastic characteristics in the circulation of cancer patients. The importance of cfNAs began to be recognized around the year 1994 [59]. At the time, the first studies on aberrant genetic alterations [194], [195], [196] and methylations [136], [161] were of high interest to the public. In 2012, a landmark study by Shaw et al. [75] showed that analyses of cfDNAs may help to detect minimal residual disease. cfDNA, cell-free DNA; cfNA, cell-free nucleic acid; ctDNA, circulating tumor DNA; NSCLC, non-small-cell lung cancer.

In solid tumors, the detection of ctDNAs is non-invasive and reproducible [30], [43], [53], [54], [55]. Plasma ctDNA was first recognized more than 60 years ago [56], representing the first step toward the development of liquid biopsies. In 1977, Leon et al. [57] detected increased concentrations of cell-free DNAs (cfDNAs) in the circulating blood of patients with lymphomas and tumors of the lungs, ovaries, uterus, and cervix using radioimmunoassay. In 1989, Stroun et al. [58] demonstrated that one-third of patients with various malignancies displayed an abundance of cfDNAs, whereas no cfDNAs could be detected in normal controls. Furthermore, it took another five years for the importance of cell-free nuclei acid (cfNA) to be recognized. Vasioukhin et al. [59] detected mutated *RAS* gene fragments in the blood of patients with myelodysplastic syndrome (MDS) and acute myelogenous leukemia (AML). In subsequent years, epigenetic aberrations in cfDNAs were identified. In 2005, Fujiwara et al. [60] demonstrated the presence of aberrant methylations on the promoters of five tumor-suppressor genes in the serum DNAs of patients with lung cancers. Within the past decade, large cohorts of studies have focused on the detection of ctDNAs in multiple types of tumors, such as cancers of the breast, colorectal region, prostate, lungs, and pancreas [61], [62], [63] (Figure 1). The details of the progress achieved by these studies would be discussed further in a later section.

In cancer patients, ctDNAs represent a variable fraction of cfDNAs (ranging from 0.01% to more than 50%) [64]. Several studies have hypothesized that ctDNAs are produced via the release of nucleic acids during the apoptosis or necrosis of cancer cells or from tumor-derived exosomes [30]. In 2015, Sun et al. found that most cfDNAs in healthy people originate from the bone marrow [65]. The average length of cfDNAs in the blood of healthy people is 70–200 bp with concentrations ranging 0–100 ng/ml. However, ctDNAs from patients with malignant tumors have lengths ranging from 200 to more than 1 kb [30], [66]. The half-lives of ctDNAs range from 15 min to a few hours, and ctDNAs are removed by the liver and kidney [67].

Novel, non-invasive applications of liquid biopsies are transforming cancer research by enabling the accurate and reliable detections of ctDNAs in plasma and urine [12]. The percentage of detectable ctDNAs depends on distinct stages (49%–78% in localized tumors and 86–100% in metastatic tumors) [68]. The sensitivity of ctDNAs can enable detections of quantitative mutations in blood plasma [30], [68]. These ctDNAs are important emerging biomarkers in cancer diagnostics, and provide non-invasive diagnostic tools for identifying cancer relapses by detecting the dynamic qualitative and quantitative changes in ctDNAs in different stages of cancer.

## Plasma ctDNA in the clinic

Monitoring of DNA mutations and epigenetic alterations presents two avenues to the detection and tracking of ctDNAs. Specific genetic variations in cancer cells can reflect the physical conditions and treatment responses of patients. Detecting DNAs with tumor-specific mutations in the peripheral blood of patients with malignancies may help to identify dynamic changes in cancer cells [69]. The ctDNA content varies in different tumor types and stages, and the mutation profiles for individual tumors may vary between patients [30], [70], [71], [72]. Unlike genetic alterations, methylation of ctDNAs is very consistent in cancer patients [14]. The aberrant methylations of ctDNAs have been described and investigated for clinical applications in most cancer types. The compositions of ctDNAs can be distinguished by analyzing the previously established methylation patterns. Under dissimilar conditions, the comparison of clinical sensitivities of detection across different studies presents enormous challenges. These conditions include the variabilities in methods of detection, number and types of targeted molecular alterations, tumor types and stages, and preselections of patients [73]. The applications of cfDNA assessments for the early diagnoses of cancers have been described extensively in previous reviews [10], [11], [12], [30]. We have illustrated previous literature on this topic published up to 2016, with emphasis on reports published between 2011 and 2016.

### Breast cancer

Breast cancer is the leading cause of cancer-related deaths in women worldwide [74]. Over the past few years, an increasing number of researchers have attempted to utilize the blood-borne biomarkers of breast cancers for the early diagnoses and precise staging of tumors, and the monitoring of treatments in patients.

In the earlier half of 2016, Shaw et al. [75] directly compared the mutational profiles of CTCs and cfDNAs from the same patients with metastatic breast cancers (MBCs). From among 112 patients, they identified five patients with more than 100 CTCs and compared these CTCs with matched cfDNAs. Unlike the levels of carbohydrate antinegen 15-3 (CA15-3) and alkaline phosphatase (ALP), total cfDNA levels and cell counts were both significantly associated with overall survivals, suggesting that cfDNAs might reflect the persisting EpCAM-positive CTCs in patients with high CTC counts. This was not the first study that quantitatively compared ctDNAs and CTCs in the circulation of individual patients. In a larger scale study comprising 640 patients that was published in 2014, Bettegowda et al. [68] demonstrated that ctDNAs were detectable in more than 75% of patients with advanced diseases, including breast cancers, and in 50% of patients with localized tumors. However, there were no cases wherein ctDNAs were absent but CTCs were detected. In contrast, in many cases wherein ctDNAs were detected (13 of 16 cases; 81.25%), no CTCs were detectable with the identical assay. Interestingly, five of these cases were of patients with breast cancers, indicating that ctDNA detection is likely to be more sensitive than CTC detection in breast cancer.

Traditional detection methods, such as immunohistochemistry and fluorescence *in situ* hybridization (FISH), have limited capability for assessments of breast cancer especially the *HER2* status [76]. Therefore, more effective methods for evaluating cancer status need to be devised. In 2012, Higgins et al. [77] screened for *PIK3CA* mutations in serum samples by beads, emulsification, amplification and magnetics (BEAMing) and reported sensitivities of 100% (14 of 14 patients). In 2013, Dawson et al. [53] described that ctDNAs are informative, inherently specific, and highly sensitive biomarkers for MBCs. Using microfluidic digital PCR and direct plasma sequencing, they were able to detect ctDNAs and CTCs in 29 (97%) and 26 (87%) of 30 women, respectively. Moreover, there existed stronger correlations between levels of ctDNAs and changes in tumor burdens than CTCs. In the meantime, Gevensleben et al. [78] detected the amplification of *HER2* in ctDNAs of patients with MBCs by using digital PCRs. Seven of 11 (64%) patients with *HER2*-amplified cancers were classified as plasma digital PCR *HER2-*positive. In 2014, Beaver et al. [79] demonstrated that pre-surgical ctDNAs with *PIK3CA* mutations from breast cancer patients showed sensitivities of 93.3% and specificities of 100% by droplet digital PCRs (ddPCRs). Also using ddPCR method, Chu et al. showed that ctDNAs from patients with MBCs displayed high frequencies of *ESR1* that encodes estrogen receptor 1. In 6 of 12 patients (50%), and seven mutations in the ctDNA of *ESR1* were detected [80]. Several other studies also showed that the detection of ctDNAs is a surrogate procedure for the traditional biopsies for breast cancer detection [81], [82], [83], [84].

Aberrant ctDNA methylation offers a more consistent and broadly applicable marker of tumor DNA in serum in comparison to DNA mutations. It has been shown that methylated *RARB2* was reduced in blood of patients following surgical removal of the tumor [85]. Additional studies also reported that methylated *RASSF1* in cancer patient can serve as an indicator of response to tamoxifen treatment [86]. However, the aberrant methylation of ctDNAs (in clinical use) in patients with breast cancers remains to be further validated [87], [88], [89], [90], [91], [92].

### Colorectal cancers

Colorectal cancers (CRCs) are a major health burden with a disease-specific mortality of about 33% [93]. Approximately 50% of the cases with CRCs are first diagnosed in late stages and about 50% of patients experience distant metastases. Thus, it is becoming increasingly urgent to develop new biomarkers for the early detection of CRCs. Some tumor-linked genetic alterations, such as in *EGFR*, *BRAF*, *ALK*, *KIT*, *PDGFR*, *HER2*, and *KRAS* [54], [94], [95], were only detected via ctDNA-based assays. Nonetheless, translating these laboratory findings into cancer therapy remains to be validated.

In 2012, Spindler et al. [96] demonstrated the expression levels of *KRAS* mutant alleles in the plasma of patients using CRCs. *KRAS* mutations were detected in 41 patients with primary or metastatic tumors and the levels of the plasma mutant *KRAS* (pmKRAS) were lower than 75%. Similar results were observed in another study by the same group [97] when examining 64 patients who were treated with temsirolimus, either alone or in combination with irinotecan. Additionally, the use of a biomarker panel consisting of three genes (*KRAS*, *TP53*, and *APC*) enabled the detection of at least one gene mutation from approximately 75% of CRC tissues [98]. In 2015, Tie et al. [99] reported that the patient-specific candidate mutations, such as KRAS G13D, in CRC tissues were detectable in the cfDNAs from 48 of 52 patients (concordance, 92.3%). In the same year, Kidess et al. [100] developed a novel assay named the sequence-specific synchronous coefficient of drag alteration (SCODA). Using the SCODA assay, they demonstrated that the detected mutations were concordant between tissues and plasma in 93% of metastatic patients (n = 38) and 54% of non-metastatic patients. Additionally, the analysis of circulating mutant DNAs has been useful in monitoring of patients receiving anti-*EGFR* therapies [94], [101], [102], [103], [104]. Several studies have also established that the mutations of beta-catenin DNA were specifically associated with CRC tissues [105], [106], [107], suggesting that the mutations of ctDNAs could serve as promising targets for the detection of CRCs.

Regarding the aberrant methylations of ctDNAs, the hypermethylation of *SEPT9* encoding septin 9 is highly associated with the progression of CRCs. Powrozek et al. [108] showed that the test for *SEPT9* methylation correctly identified lung cancers in 31 of 70 (44%) samples, whereas positive results were only detected in 4 of 100 controls. In addition, hypermethylated *RASSF1A* and *E-CAD* have been considered as new biomarkers for CRCs as well [109], [110].

### Non-small-cell lung cancers

Tumor tissue genotyping is used routinely in cases of lung cancers to identify specific and targetable oncogenic alterations, including *EGFR* mutations and *ALK* rearrangements. In 2015, Alecensa (alectinib) and Tagrisso (osimertinib) were approved by the US Food and Drug Administration (USFDA) for personalized treatment of non-small-cell lung cancers (NSCLCs). In the past few years, studies have reported a series of achievements relevant to *EGFR* mutations [111], [112], [113]. The cobas® *EGFR* Mutation Test v2 from Roche was the first liquid biopsy test approved by the USFDA in June 2016 for the detection of *EGFR* exon 19 deletions or exon 21 (L858R) substitution mutations of NSCLC patients. More information can be found at http://www.fda.gov/drugs/informationondrugs/approveddrugs/ucm504540.htm. This represents great progress for the clinic utility of ctDNAs.

The sensitivities of detecting ctDNAs in NSCLC patients varied in different studies. For example, ctDNAs were detected in 7 of 8 (88%) patients with stage III disease [114], 1 of 1 (100%) with stage III, 4 of 4 (100%) with stage IV [115], and 4 of 5 (80%) with stage IV [68]. Therefore, studies on larger cohorts are needed for more reliable results.

In addition, the hypermethylation of 80 genes have been observed in lung cancers, and the methylation levels show spatial–temporal specificities [116].

### Other types of tumors

A previous study showed that a high proportion of DNAs from cancerous tissues were found in the plasma of 29 patients with liver cancers. About 24% of the plasma DNAs were from the liver, whereas only 10.7% plasma DNAs were from the liver in the control group [65]. In addition, the sensitivities of detecting ctDNA mutations in primary pancreatic cancers are usually 30%–50%, and the specificities are usually higher (approximately 90%) [117]. More studies on other types of cancers, specifically prostate cancers [41], [68], [118], [119], [120], ovarian cancers [121], [122], and pancreatic carcinomas [123], were still underway. The deconvolution of methylations showed that the median percentage of cfDNA present in the plasma from patients with hepatocellular carcinomas (HCCs) and control subjects were 24.0% and 10.7%, respectively [65]. A summary of DNA methylation for cancer detection is shown in Table 2.

Our understanding of DNA methylations in cancers has been deepened during the last three decades [124], [125]. Circulating methylated DNAs have been considered as potential biomarkers for the detection of several cancers, including colorectal, lung, breast, and pancreatic cancers. The methylation of DNA that encodes tumor suppressors and metastases suppressors can be used to determine tumor proliferation and metastases in CTCs [15], [16], [17], [18]. Unlike DNA mutations, the aberrant methylation of specific promoter regions that typically occurs at multiple sites may be a consistent characteristic of cancers [17], [126]. Such consistency makes methylation of ctDNAs a great choice when designing broadly-applicable clinical assays [14], [65]. Small differentially-methylated regions (DMRs) such as CpG island shore methylations and large blocks (hypomethylated blocks), appear at early stages. They can potentially be used as biomarkers for early cancer detection [127].

## Methods for detecting ctDNAs

### Mutations of ctDNAs

The quantity of detectable ctDNAs depends on the tumor burden and type, as well as other potential biological mechanisms, such as the activities of plasma nucleases [137], [138]. It is necessary to understand the technical aspects of ctDNA detection, in order to evaluate the clinical value of current studies on ctDNAs. Due to recent technological advances, many methods are now available for detecting genetic mutations in cancers. Methods such as microarray-based comparative genome hybridization (CGH), single nucleotide polymorphism (SNP) analysis, and next-generation sequencing (NGS) have been adopted to tackle with the issue of sensitivity and accuracy for the detection of tumor genomes, and have yielded crucial insights into tumor biology [55]. In addition, array-based CGH and SNP arrays, both based on the hybridizations with the arrays of oligonucleotide probes immobilized on a slide, allow for the identification of genetic variations as well. The SNP arrays contain unique nucleotide sequences used as probes that hybridize with fragmented single-stranded DNAs (ssDNAs). Furthermore, genome-wide association studies (GWAS) facilitate the analyses of more than one million SNPs via the chip-based microarray technology, and open up the possibility of detecting tumor-specific DNAs for the development of blood-based diagnostic tests for cancers [139], [140]. There is an urgent need to update related technologies for detecting low levels of tumor DNAs in the circulation of patients with cancers (Table 3).

More cutting-edge technologies have emerged, resulting in the incorporation of higher sensitivities and personalized therapies for cancers. For instance, targeted plasma re-sequencing (TAm-Seq) was first used in 2012 to detect *de novo* mutations [140]. TAm-Seq could identify *TP53* mutations by tracking ctDNAs in ovarian cancer patients [140]. For personalized healthcare, the massively parallel sequencing (MPS) technique, including the personalized analysis of rearranged ends (PARE) and shotgun MPS, can track alterations in ctDNA levels in patients before and after surgery [114]. In 2013, using shotgun MPS, Chan et al. identified single nucleotide variants (SNVs) and copy number variations (CNVs) from the plasma of four HCC patients [146]. In addition, many other methods for detecting ctDNAs are also available, such as allele-specific PCR-based methods, digital PCR-based methods, whole-genome sequencing (WGS), and whole-exome sequencing (WES). A comparison of these methods is presented in Table 3 [4].

In 1999, Vogelstein et al. described an approach termed digital PCR, on the basis of the traditional PCR, and analyzed mutations using fluorescent probes [147]. Using digital PCRs, they can detect a small number of mutant cells among several normal cells with a lower signal-to-noise ratio. ddPCR in combination with digital PCR and rapid microfluidic analysis can significantly increase the sensitivities of ctDNA detection. Compared to real-time qPCRs (RT-qPCRs), ddPCRs exhibits higher precision and can absolutely quantify nucleic acids [142]. Several studies have reported ddPCR as a method for ctDNA detection in several cancers, including breast cancers [72], [80], melanomas [148], [149], HCCs [150], and colorectal cancers [151], [152]. This technique may serve as a potential surrogate for ctDNA detection, and has exhibited great potential for clinical applications.

In addition, Alizadeh and Diehn developed a novel method, termed cancer personalized profiling by deep sequencing (CAPP-Seq). It is a capture-based NGS method for the detection of ctDNAs [143] and ultrasensitive for the quantification of ctDNAs. Application of CAPP-Seq in NSCLC samples lead to the identification of mutations in more than 95% of tumors [143]. For 100% of stage II–IV and 50% of stage I patients with NSCLCs, ctDNAs showed a specificity of 96% for mutant allele fractions down to approximately 0.02%. This new strategy can examine large portions of the genome for early cancer detection [143], [144]. In March 2016, Newman et al. [145] developed a novel method called integrated digital error suppression-enhanced cancer personalized profiling by deep sequencing (iDES-enhanced CAPP-Seq). With a sensitivity of 92% and a specificity of more than 99.99% for the variant alleles, and concurrently, a sensitivity of 90% and a specificity of 96% in patients, iDES-enhanced CAPP-Seq enabled the biopsy-free profiling of mutations in the EGFR kinase domain [145] (Table 3). As mentioned above, these techniques enable ctDNA analysis to track the tumor burden without conducting sequentially repeated biopsies.

### ctDNA methylation

Strategies used in DNA methylation analysis can be classified into two groups: site-specific and genome-wide methylation detections. Currently, detection of site-specific methylations of DNA has been conduced more often [14], [153], [154], [155]. Many methods originally used for site-specific detection of genomic DNA methylations are suitable for site-specific detection of ctDNA methylations as well. Following bisulfite conversion or methylated ctDNA enrichment, detection of methylated ctDNAs can be facilitated via different PCR amplification methods, including the conventional methylation-specific PCR (MSP) [156], [157], [158], quantitative multiplexed methylation-specific PCR (QM-PCR) [159], and methylation on beads (MOB) [160]. A method for the enrichment of methylated CpG sequences has also been developed for use in kits [156]. The enriched methylated DNA can be used for both sequencing and PCR amplifications. Conventional MSP can be used directly in the detection of ctDNA methylations [157], [158]. This method requires only 5 ml of peripheral blood, and can be applied in non-invasive measurements clinically [161]. Furthermore, fluorescence-based real-time MSP, an improved form of the aforementioned technique, combines the use of fluorescent probes and detection in real-time to facilitate the quantitative detection of DNA methylations [162]. Furthermore, quantum dots (QDs) have been used as fluorophores and fluorescence resonance energy transfer (FRET) donors for biological sensing and detection of biomolecular targets [163]. Another modified version of conventional PCR, namely MOB, integrates three processes – DNA extraction, bisulfite conversion, and PCR – into a single tube by using silica superparamagnetic beads as DNA carriers [160], [164]. In 2014, the cMethDNA assay, a new method based on the standard QM-MSP, was reported for the identification of novel methylated breast cancer genes in serum. Owing to its ability of enhancing methylation signals, cMethDNA assay would be suitable for detection of ctDNA methylations [165].

Although there are many bisulfite conversion- and enrichment-based methods for genomic DNA methylation analysis, few of these methods can be applied in ctDNA methylation analysis, owing to the characteristics of ctDNAs. For example, bisulfite conversion-based methods, including MethylC-Seq or BS-Seq, are not suitable for this purpose because whole-genome bisulfite sequencing requires relatively large samples of DNAs [166] to be subjected to the sodium bisulfite-mediated conversion of unmethylated cytosines, and obtaining the sequence information through computing conversion ratio [167]. A method called reduced representation bisulfite sequencing (RRBS) was developed with a focus on CpG islands and promoter regions, allowing the sequencing of methylated regions that are otherwise unable to be properly profiled using conventional bisulfite sequencing techniques [168]. In addition, studies employing enrichment-based methods, including methylated DNA binding domain-sequencing (MBD-Seq) [169] and methylated DNA immunoprecipitation sequencing (MeDIP-Seq) [170], have not been reported for use at low concentrations (<150 ng) of DNA [171]. Both MBD-Seq and MeDIP-Seq are based on DNA enrichment technologies, which utilize methyl-CpG binding domain protein 2b and 5-methyl cytosine antibodies, respectively, may exhibit sensitivities lower than single-base resolution [169], [170].

In recent decades, an increasing number of studies have focused on the potential use of ctDNA methylations as biomarkers for early detection of cancers, for cancer screening, and for monitoring the efficacies of anticancer therapies [172], [173]. Shot-gun massively parallel bisulfite sequencing, another bisulfite sequencing-based technique, was also developed for the detection of ctDNA methylations. This method detects ctDNAs with high sensitivity and specificity, even at a low sequence depth. Additionally, the volume of sample used in this method can be reduced to 4 ml of plasma [172].

In 2015, Wen et al. [173] explored a genome-wide methylated CpG tandem amplification and sequencing (MCTA-Seq) method to detect hypermethylated CpG islands in ctDNAs. Given ctDNA is fragmented and accounts for only a small portion of cfDNA, this sensitive method is suitable for detecting ctDNA methylation, since it only requires small amounts of ctDNA (as low as 7.5 pg) [173]. This is the first genome-wide technique developed for the detection of ctDNA methylations (Table 4).

## Challenges in circulating DNA for early diagnosis

Although tumor-specific mutations and methylations in ctDNAs are potential targets for the non-invasive cancer detection, and for the diagnoses, prognostic management, and guidance for treatments of these cancers, there are still barriers in the accurate detection of specific cell-free nucleic acids [175]. Common biomarkers for all types of tumors have not been discovered yet.

Notably, there is no unified standard for detection. There is no consensus yet regarding the typical concentrations of cfDNAs present in the blood of healthy people. Since steps involving ctDNA extraction are not always described in detail in published studies, the concentrations of ctDNA detected tend to vary a lot [67], [120], [176], [177], [178], [179]. Based on current situation, a large blood sample is needed for the detection of ctDNAs due to their low blood concentrations [180], [181], [182], [183]. The rate of purification of these samples needs to be improved greatly. Tumor DNA fragments are diluted with normal DNA in circulation, which may hamper subsequent analysis. Furthermore, false-positives still occur in several approaches due to the inadequate sensitivities and specificities of these techniques [4]. Technological improvements and a better understanding of ctDNA biology are necessary to achieve better outcomes. Techniques using ctDNA are limited in the clinical setting because of their high costs, and the clinical use of this technique is still a subject of debate due to the needs to further improve the accuracy and sensitivity of ctDNA detection.

ctDNA has the advantage and the potential of serving as a relatively stable biomarker. To develop clinically valuable biomarkers, close collaborations are needed between the clinicians and scientists. In the context of strong public expectation and the increasingly-established biotechnology companies as “cancer detection from a drop of blood”, liquid biopsy is perhaps overestimated to some extent. Diagnostic kits that are reliable and exhibit both higher sensitivities and specificities are therefore overdue. However, before translating basic research into clinical utility for precise treatment, it is prerequisite to gain in-depth mechanistic understanding on how heterogeneous cancer cells in different cancers behave at various stages, rather than merely improving the methods of detection.

To achieve the goal of PM, we would need to select the optimal strategies or drugs accordingly to the characteristics of patients, based on the available early detection and screening for cancer patients before initiation of treatments. However, due to the lack of subsequent treatment strategies or drugs, we have not benefitted much from the head start provided by PM for early detection of cancers, even though we have screened for several risk factors related to the cancers. Therefore, we need to discover and identify new targets in order to develop more effective drugs.

In the context of precision medicine, the construction of a comprehensive, accurate, and multi-dimensional cancer genomic-epigenetic map remains a challenge. Additionally, lack of timely and efficient communication between researchers and clinicians poses further challenges. A better appreciation of the patients’ needs by basic scientists, and a clearer understanding of the research progress and the potential clinical applications of the basic research by clinicians would help bridge the gaps.

## Opportunities on the path to clinical utility

Given the well-recognized difficulties in repeatedly obtaining tissue biopsies, liquid biopsy can be an effective method for cancer detection. Individual patients with either metastatic or primary tumors may vary in their genomic, epigenetic, and transcriptomic compositions [30], [184]. Liquid biopsies help to overcome the problems of inherent tumor heterogeneity of tissue biopsies for personalized therapy, and can be used for detecting cancers even before diagnosis by imaging studies [185]. Despite the challenges in the clinical application of ctDNAs, their detection can be conducted repetitively in real-time, conferring the advantages of being non-invasive, non-injurious, as well as highly sensitive and specific. Also, ctDNAs might hold more promise as biomarkers, compared with protein biomarkers, as the ctDNAs may be more informative, accurate, and specific [53], [68]. In addition, technological advances facilitate the detection of thousands of CpG sites and whole-genome sequences, which can be applied to bisulfite converted ctDNAs and used for cancer detection as well [186], [187]. As the identification of DMRs becomes easier, they are likely to be used as cancer biomarkers [14], [15], [72], [108]. Furthermore, the cost of sequencing was $100,000 per genome in 2009, but fell remarkably to below $1500 by late 2015 (http://www.genome.gov/sequencingcosts/).

Targeted re-sequencing of cfDNAs can be performed to elucidate mutations in serum samples. In-depth re-sequencing and digital PCR [188] analyses enables more sensitive detection and monitoring of specific mutations in minute amounts of ctDNA [189]. It is expected that NGS will lower the overall costs, speed up the turnaround times, increase the detection sensitivities, as well as enable the detections of rare gene mutations and individual epigenetic markers in the near future. Additionally, in the clinic, NGS may become applicable to CTCs and cfDNAs in plasma. Targeted NGS of ctDNAs has the potential clinical utility of enabling early diagnoses of tumors and implementation of targeted therapies. This would create more opportunities for the discovery of cancer biomarkers and improve the sensitivity and specificity of such detection. This new task also requires cooperation among the leading research groups and investment from pharmaceutical and biotechnology companies.

Currently, the USFDA and the China FDA (CFDA) have certificated some DNA methylation-based biomarkers, including genes encoding NDRG family member 4 (*NDRG4*) and bone morphogenetic protein 3 (*BMP3*; USFDA approval in 2014) [190], *SEPT9* [191], and the gene encoding short stature homeobox 2 (*SHOX2*; CFDA approval in 2015) [192], which give a boost to ctDNA detection on larger scales. Additionally, in 2016, the *EGFR* Mutation Test v2 (cobas) [193] became the first US FDA-approved in-vitro diagnostic (IVD) medical device for liquid biopsy for detecting *EGFR* mutations in NSCLCs. Biocept, Guardant Health, Neo Genomics Laboratories, Qiagen, and many other US-based companies have provided ctDNA detection services to their consumers. Founded by Illumina, a new company, named Grail, aims at developing liquid biopsy-based tests that would cost less than $1000 per test, and is recruiting the best talents in the field of cancer detection. Their products are planned to reach consumers by the year 2019 (http://www.grailbio.com/). The biotechnology companies in China also have invested in this emerging field; these companies include Surexam, Annroad, BGI, *etc*. The development trend and the current progress in the field afford more opportunities than challenges for liquid biopsy detection. From 2015 to 2016, nearly thirty kinds of new anti-cancer drugs were approved by the USFDA, such as Tecentriq (atezolizumab, Genentech, US), Tarceva (erlotinib, Astellas Pharm, US), Alecensa (alectinib, Hoffmann-La Rocha, Swiss) for treatment of NSCLCs, and Lenovima (lenvatinib, Eisai, Japan) and Cabometyx (cabozantinib, Exelixis, US) for the treatment of renal cell carcinomas (RCCs). The highly-sensitive ctDNA detection could be harnessed by the newly-approved targeted therapies and precise treatments and ultimately benefit the patients.

Monitoring of cancers by measuring ctDNA dynamics in blood or serum is a new and developing area of research. Based on the current research progress and the growth of the medical industry, we believe that ctDNA assays may be used to personalize treatments real-time for cancer patients in the future, based on their individual ctDNAs or ctDNA methylation levels, for diagnoses, prognoses, and guidance for treatments. However, there is much room for improvement before this technology can be routinely applied in clinical settings.

## Competing interests

The authors declare that they have no conflict of interests.

## Figures and Tables

**Table 1:** **Comparison of different cancer detection methods for their clinical utilities**

**Detection method**		**Strengths**	**Limitations**	**Refs.**
Imaging-based methods (CT, MRI, PET, *etc*.)		Rapid; easy to use; displaying solid tumor visually	Unable to detect minimal residual disease; exposing patients to additional ionizing radiation	[19], [20], [21]

Solid biopsy		Reflecting certain histological issues; short operating time	Unable to represent the entre tumor due to the intra- and inter-tumor heterogeneity; serial biopsy often impractical; discomfort suffered by the patient; not accessible for some tumors	[22], [23], [24], [25]

Liquid biopsy	Protein (CA-125, CEA, PSA, *etc*.)	Non-invasive; easy to obtain	Low specificity; Unable to be detected in vast majority of patients with advanced cancers	[26], [27], [28]
	CTCs	Non-invasive; high specificity; demonstrating colocalization of signals; evaluating protein expression; potentially addressing tumor heterogeneity	Low signal-to-noise; affected by heterogeneity on selection methods	[7], [11], [29]
	ctDNA	Non-invasive; high specificity and sensitivity; providing personalized snapshot of disease; fully representing tumors	Low signal-to-noise; lack of colocalization, protein expression, and functional studies	[10], [30], [31]
	Circulating cfRNA	Non-invasive; stable; demonstrating distinct gene expression patterns from particular tumor	Lack of large-scale studies; lack of correlations between tumor behavior and findings	[32], [33], [34]
	Exosomes	Non-invasive; stable within exosomes; easy to isolate or enrich	Lack of large-scale studies; hard to define	[35], [36], [37]

*Note*: CT, computed tomography; MRI, magnetic resonance imaging; PET, positron emission tomography; CA-125, carcinoma antigen-125; CEA, carcinoembryonic antigen; PSA, prostate-specific antigen; CTC, circulating tumor cell; ctDNA, circulating tumor DNA; cfRNA, cell-free RNA.

**Table 2:** **The DNA methylation for cancer detection**

**Cancer type**	**Marker**	**Sensitivity**	**Specificity**	**Refs.**
Colorectal cancer	*MLH1*	3/18 (17%)	N/A	[128]
	*CDKN2A* (*INK4A*)	14/52 (27%)	44/44 (100%)	[129]
	*ALX4*	21/58 (36%)	N/A	[130]
	*CDH4*	25/30 (83%)	36/52 (70%)	[131]
	*NGFR*	32/46 (70%)	17/17 (100%)	[127]
	*RUNX3*	68/133 (51%)	150/179 (84%)	[132]
	*SEPT9*	11/17 (65%)	10/10 (100%)	[133], [134]
	*TMEFF2*	87/133 (65%)	123/179 (69%)	[132]

Breast cancer	*CDKN2A* (*INK4A*)	5/35 (14%)	N/A	[135]

Lung cancer	*CDKN2A* (*INK4A*)	3/22 (14%)	N/A	[136]
	*DAPK1*	4/22 (18%)	N/A	
	*GSTP1*	1/22 (5%)	N/A	

*Note*: Table was adapted from Jin et al. [126] with permission. MLH1, mutL homolog 1; CDKN2A (INK4A), cyclin dependent kinase inhibitor 2A; ALX4, ALX homeobox 4; CDH4, cadherin 4; NGFR, nerve growth factor receptor; RUNX3, Runt related transcription factor 3; SEPT9, septin 9; TMEFF2, transmembrane protein with EGF-like and two follistatin-like domains 2; DAPK1, death associated protein kinase 1; GSTP1, glutathione *S*-transferase Pi 1.

**Table 3:** **Comparison of methods for ctDNA detection**

**Method**	**Description**	**Detection limit (% ctDNA)**	**Strengths**	**Limitations**	**Refs.**
Allele-specific PCR	Preferentially amplifying rare mutant DNA molecules	0.10–1.00	Ease to use; lowest cost	Lower sensitivity; only able to test small number of genomic positions in a sample	[141]

Digital PCR	Counting mutant molecules via partitioning of DNA molecules	0.01	High sensitivity	Only able to test small number of genomic positions in a sample	[142]

NGS amplicon based	Deep sequencing of PCR amplicons	0.01–2.00	High sensitivity (some methods); less expensive than other NGS methods	Less comprehensive than other NGS methods; unable to detect SCNAs; unable to detect rearrangements without assay customization	[55]

WGS	Deep sequencing of entire genome	1.00	Interrogating entire genome; broadly applicable without personalization	Expensive; low sensitivity; mostly limited to SCNA detection	[41]

WES	Deep sequencing of exome	5.00	Interrogating entire exome; broadly applicable without personalization	Expensive; low sensitivity	[41]

CAPP-Seq	Targeted hybrid capture	0.01	High sensitivity for SNVs, indels, rearrangement, and SCNAs detection; broadly applicable without personalization	Less comprehensive than WGS or WES	[143], [144]

iDES-enhanced CAPP-Seq	Targeted hybrid capture and integrated digital error suppression	0.01	High scalability, flexibility, and coverage uniformity; able to reliably evaluate all mutation classes in a single assay	Less comprehensive than WGS or WES	[145]

*Note*: Table was adapted from Chaudhuri et al. [4] with permission. ctDNA, circulating tumor DNA; SCNA, somatic copy number alteration; SNV, single nucleotide variation; WES, whole-exome sequencing; WGS, whole-genome sequencing; CAPP-Seq, CAncer personalized profiling by deep sequencing; iDES, integrated digital error suppression.

**Table 4:** **Methods of detection of DNA methylation in circulating cells**

**Detection type**	**Method**	**Description**	**Refs.**
Site-specific detection	Conventional MSP	Requiring a sample spot (5 ml of peripheral blood); Able to be used in the detection of certain methylated genes in the plasma of serum; using specific PCR primers for methylated sequences	[156], [157], [158]
	Fluorescence-based real-time MSP	Facilitating quantitative detection; sensitive; requiring prior knowledge of the methylated sequences	[174]
	QDs-FRET	Able to reduce the background for detecting targets at low concentration; greater sensitivity; limited FRET efficiency; impractical for challenging samples such as serum and plasma	[163]
	MOB	Easy to handle; increased detection throughput; providing efficient, sensitive methylation detection in diagnosis; able to be used in blood samples	[160], [164]
	cMethDNA	High sensitivity, specificity, reproducibility, dynamic range, and quantitative advantages; detecting methylated site at low levels in cell-free circulating serum DNA; promising new liquid biopsy tool	[165]
Genome-scale detection	Conventional bisulfite conversion-based methods	Gold standard for the detection of DNA methylation; requiring a relatively large amount of sample; focused on CpG islands or promoter regions	[166], [167], [168]
	Conventional enrichment-based methods	No conversion treatment; requiring a high concentration of DNA; likely ignoring other methylated sites when using antibody against 5 mC or 5 mCG	[169], [170], [171]
	Short-gun massively parallel bisulfite sequencing	Detecting with high sensitivity and specificity even at a low sequence depth with 10 million sequencing data; requiring 4 ml plasma only	[172]
	MCTA-seq	Working well with ctDNA samples as small as 7.5 pg; able to simultaneously detect thousands of hypermethylated CpG islands in cfDNA	[173]

*Note*: MSP, methylation-specific PCR; QDs-FRET, quantum dots-fluorescence resonance energy transfer; MOB, methylation on beads; MCTA-seq, methylated CpG tandem amplification and sequencing; ctDNA, circulating tumor DNA; cfDNA, cell-free DNA.
